# A Food-Based Sequential KETOgenic Nutritional Protocol for Mediterranean Diet Non-Responders with COMPlicated Obesity: A Real-World Observational Study (KETOCOMP)

**DOI:** 10.3390/nu18182982

**Published:** 2026-09-11

**Authors:** Maurizio Romano, Pauline Celine Raoul, Gianluca Ianiro, Debora Rondinella, Eleonora Ribaudi, Francesca Sofia Galli, Marco Cintoni, Antonio Gasbarrini, Esmeralda Capristo, Maria Cristina Mele, Emanuele Rinninella

**Affiliations:** 1 Digestive Disease Center (CEMAD), Department of Medical and Surgical Sciences, Fondazione Policlinico Universitario Agostino Gemelli IRCCS, Largo A. Gemelli 8, 00168 Rome, Italy; maurizio.romano@guest.policlinicogemelli.it (M.R.); gianluca.ianiro@policlinicogemelli.it (G.I.); debora.rondinella@unicatt.it (D.R.); eleonora.ribaudi@policlinicogemelli.it (E.R.); antonio.gasbarrini@unicatt.it (A.G.); 2 Clinical Nutrition Unit, Department of Medical and Surgical Sciences, Fondazione Policlinico Universitario Agostino Gemelli IRCCS, Largo A. Gemelli 8, 00168 Rome, Italy; paulineceline.raoul@policlinicogemelli.it (P.C.R.); f.sofiagalli@gmail.com (F.S.G.); marco.cintoni@unicatt.it (M.C.); mariacristina.mele@unicatt.it (M.C.M.); 3 Department of Translational Medicine and Surgery, Università Cattolica del Sacro Cuore, 00168 Rome, Italy; esmeralda.capristo@unicatt.it; 4 Human Nutrition Research Center, Università Cattolica del Sacro Cuore, 00168 Rome, Italy; 5 Obesity Disorders Unit, Department of Medical and Abdominal Surgery and Endocrine-Metabolic Sciences, Fondazione Policlinico Universitario Agostino Gemelli IRCCS, Largo A. Gemelli 8, 00168 Rome, Italy

**Keywords:** complicated obesity, metabolic syndrome, ketogenic diet, very low-calorie ketogenic diet, weight loss, body composition, fat-free mass, body cell mass, phase angle, mediterranean diet non-responders, KETOCOMP

## Abstract

**Background**: Obesity is a complex, chronic disease with metabolic complications. Many patients respond suboptimally to conventional interventions like the hypocaloric Mediterranean diet (MD), highlighting the need to investigate alternative supervised nutritional strategies in patients with an insufficient response to conventional dietary treatment. **Objectives**: This study aimed to evaluate the KETOCOMP (Food-Based KETOgenic Nutritional Protocol for Patients with COMPlicated Obesity), a medically and dietitian-supervised, food-based sequential ketogenic nutritional protocol consisting of a ketogenic phase followed by controlled carbohydrate reintroduction, for improving anthropometric and body composition in patients with complicated obesity who were non-responsive to conventional dietary treatment. **Methods**: In this longitudinal, observational study, adult patients with obesity/overweight and metabolic comorbidities, previously non-responsive to a hypocaloric MD, were identified. Patients followed a 4-week food-based ketogenic diet (approx. 1200 kcal/day, <20 g carbohydrate, CHO) followed by a 4-week low-carbohydrate reintroduction phase (approx. 1300 kcal/day, up to 100 g CHO). Anthropometric and body composition parameters were assessed at baseline (T0), after the MD (T1), after the ketogenic phase (T2), and after the low-carbohydrate phase (T3). Intra-subject comparisons were performed. **Results**: Across the entire observed nutritional pathway (T0–T3), body weight (BW), body mass index (BMI), and fat mass (FM) decreased by 7.7%, 9.45%, and 23.1%, respectively. Using T1 as the relevant baseline for the second-line KETOCOMP sequence, mean BW and FM decreased by 7.79 kg and 6.29 kg, respectively, from T1 to T3; during the ketogenic phase alone (T1–T2), BW decreased by 6.10 kg and FM by 4.80 kg. Body Cell Mass (BCM) and Phase Angle (PhA) showed no statistically significant changes during the ketogenic and carbohydrate-reintroduction phases, whereas Fat-Free Mass (FFM) decreased significantly. Significant reductions in waist and hip circumferences (WC, HC) were also observed. No serious adverse events were documented among included participants; however, discontinuation rates could not be assessed because completion through T2 formed part of cohort selection. **Conclusions**: The KETOCOMP protocol may represent a clinically applicable second-line nutritional strategy for patients with obesity and metabolic complications who respond suboptimally to conventional dietary treatment. The observed reductions in body weight and fat mass, together with the absence of significant changes in BCM and PhA, suggest a favorable short-term change in body composition. However, larger prospective controlled studies incorporating standardized clinical and biochemical safety monitoring and direct assessment of skeletal muscle are required to confirm these findings and evaluate longer-term outcomes.

## 1. Introduction

Obesity has emerged as one of the 21st century’s most pressing and intricate global health challenges, imposing a substantial burden on healthcare systems worldwide [1,2]. Far from being merely an aesthetic concern or an isolated risk factor, obesity is now universally recognized as a complex, chronic, and multifactorial disease characterized by excessive or abnormal adipose tissue accumulation that profoundly compromises health and quality of life [3,4,5,6]. This modern conceptualization, defining obesity as a “neurobehavioral, progressive, and relapsing, multifactorial disease,” highlights that surplus body fat frequently leads to adipose tissue dysfunction with adverse metabolic, biomechanical, and psychosocial consequences [6]. The human body actively defends a higher BW through potent homeostatic mechanisms, which can impede sustained weight loss and contribute to its chronic, relapsing nature [7,8,9,10,11,12,13,14]. Its etiology is deeply complex, arising from an intricate interplay of genetic, environmental, epigenetic, and neuroendocrine factors that extend beyond a simplistic energy imbalance [1,5,15]. The global economic impact of obesity is immense, with projections indicating it could reach approximately $4.32 trillion annually by 2035 if current trends persist [16,17].

While the BMI remains the primary diagnostic tool in clinical and epidemiological settings due to its practical simplicity [18,19,20], its limitations in assessing individual health status are well-documented. BMI does not directly quantify body fat, differentiate between fat and lean mass, nor does it precisely indicate adipose tissue distribution [2,3,21]. Crucially, abdominal adiposity is a stronger predictor of cardiometabolic risk than BMI alone [3], underscoring the need to distinguish between metabolically healthy obesity (MHO) and metabolically unhealthy obesity (MUO). Individuals classified as MHO, despite having a BMI of 30 kg/m^2^, exhibit fewer cardiometabolic abnormalities [22] and often demonstrate better adipose tissue expandability [22]. However, MHO is frequently a transient phenotype [22], with many individuals progressing to a metabolically unhealthy state, and it still carries a higher cardiometabolic risk compared to metabolically healthy normal-weight individuals [22].

The progression from MHO to MUO is driven by a complex interplay of pathogenic mechanisms centered on dysfunctional adipose tissue [4,23]. The key pillars of this pathogenesis are insulin resistance, chronic low-grade inflammation (LGCI), and lipotoxicity. Dysfunctional adipose tissue, characterized by hypertrophied adipocytes and immune cell infiltration, leads to altered adipokine secretion, increasing pro-inflammatory cytokines including tumor necrosis factor-α (TNF-α), interleukin-6 (IL-6), and resistin while decreasing anti-inflammatory ones [23,24,25]. This LGCI contributes to systemic dysfunction and insulin resistance [1,4,24]. Insulin resistance, defined as a reduced cellular sensitivity to insulin, is a central driver of type 2 diabetes mellitus (T2DM), hypertension, dyslipidemia, and atherosclerosis [26,27,28]. When adipose tissue’s storage capacity is overwhelmed, lipids accumulate ectopically in non-adipose tissues (e.g., liver, skeletal muscle, heart, pancreas), resulting in lipotoxicity and organ damage [4,23,29,30,31,32,33]. These mechanisms are deeply interconnected, forming a self-perpetuating network that accelerates organ damage and the progression of comorbidities [4,23,31].

This cluster of cardiometabolic risk factors is formally recognized as Metabolic Syndrome (MetS), which significantly elevates the risk of developing cardiovascular diseases (CVD) and T2DM [34,35]. MetS is defined by the presence of at least three of five criteria: abdominal obesity, hypertriglyceridemia, low high-density lipoprotein cholesterol, hypertension, and elevated fasting glucose [35]. Both obesity and MetS predispose individuals to a wide spectrum of comorbidities, including T2DM, atherogenic dyslipidemia [3,36], arterial hypertension [36,37,38], and Metabolic Dysfunction-Associated Steatotic Liver Disease (MASLD) [39,40]. The chronic pro-inflammatory state associated with obesity and MetS also profoundly impacts the quality of life and serves as an independent risk factor for adverse health outcomes [24,36,41,42,43].

International guidelines consistently advocate for comprehensive lifestyle interventions, including balanced hypocaloric diets and regular physical activity, as the cornerstone of obesity management [44,45]. The MD, in particular, is widely lauded for its benefits in reducing the risk of cardiovascular disease (CVD), T2DM, and MetS [46,47,48]. However, a significant percentage of patients with obesity and metabolic comorbidities exhibit a suboptimal response to these standard approaches and are thus classified as “non-responders” [49]. Reasons for this include individual biological heterogeneity (e.g., metabolic adaptation, genetic factors), challenges with long-term adherence, and persistent metabolic dysfunction (e.g., severe insulin resistance) that conventional interventions may not adequately address [49,50,51]. For patients with a clinically insufficient or suboptimal response, there is a rationale for considering structured second-line nutritional strategies [49,52,53].

In this context, the ketogenic diet (KD), an eating regimen characterized by very low carbohydrate intake, adequate protein, and high fat [54], has emerged as a promising alternative. Initially utilized for pharmacoresistant epilepsy [55,56], its application has expanded to obesity and metabolic comorbidities due to its profound physiological and biochemical effects [57,58]. The KD induces nutritional ketosis, a metabolic state where ketone bodies (primarily β-hydroxybutyrate, BHB) become the primary energy source. This promotes efficient fat oxidation, preserves lean mass, and mitigates metabolic adaptation [54,58,59]. Crucially, ketosis improves insulin sensitivity [60,61], restores metabolic flexibility, and effectively suppresses appetite through the action of ketone bodies on hunger/satiety hormones [54,62]. Furthermore, KD positively impacts dysfunctional adipose tissue, modulates inflammatory pathways (e.g., BHB inhibits the NOD-like receptor protein 3 (NLRP3) inflammasome) [63], and significantly improves lipid profiles [58]. Its efficacy in reducing intra-hepatic fat and improving liver markers makes it particularly effective for MASLD [64].

Scientific societies globally, including European and Italian bodies, increasingly recognize the KD, especially Very Low Calorie Ketogenic Diets (VLCKDs) or normoproteic ketogenic diets, as a valid and effective strategy for managing obesity and its complications [65,66,67]. While American guidelines from organizations like the American Diabetes Association also acknowledge the utility of low-carbohydrate diets for glucose and weight management [62], they consistently emphasize that KD must be a medically supervised nutritional therapy. This requires careful patient selection, personalized prescription, rigorous monitoring of clinical and biochemical parameters, and a structured transition phase to support clinical safety, minimize side effects (e.g., “keto flu”), and promote long-term success [57,65,66,68].

Given the limited response to conventional dietary treatment observed in a subset of patients with complicated obesity, the KETOCOMP protocol was developed as a structured second-line nutritional pathway rather than as an isolated ketogenic intervention. Its distinctive features include the selection of patients who had not achieved clinically meaningful weight loss after a supervised hypocaloric Mediterranean diet; a moderately energy-restricted, entirely food-based ketogenic phase; protein intake individualized according to ideal body weight; continuous medical and dietitian supervision; and a subsequent controlled carbohydrate reintroduction phase. This sequential design was intended not only to reactivate weight loss through nutritional ketosis, but also to improve body composition, monitor changes in metabolically active tissue, and facilitate the transition toward a more sustainable dietary pattern.

The present study therefore aimed to evaluate the effectiveness of the KETOCOMP protocol in adults with complicated obesity and metabolic comorbidities who had previously shown a suboptimal response to a supervised hypocaloric Mediterranean diet. Specifically, we assessed changes in body weight, anthropometric parameters, and body composition across the ketogenic and carbohydrate reintroduction phases. Evaluating this protocol is clinically relevant because evidence remains limited regarding structured second-line nutritional pathways specifically developed for patients who do not achieve clinically meaningful weight loss with conventional dietary treatment.

## 2. Materials and Methods

### 2.1. Study Design and Participants

This study was designed as a retrospective, observational, longitudinal, single-arm, intra-subject analysis conducted in an outpatient Clinical Nutrition Unit specialized in the management of Metabolic Syndrome. The protocol involved the review of clinical records from adult patients who, as part of routine clinical practice, underwent a structured sequential nutritional pathway.

Each participant served as their own control, with evaluations performed at four time points corresponding to sequential phases of a nutritional protocol: T0 (baseline), T1 (post–MD), T2 (post–KD), and T3 (post–low carbohydrate reintroduction diet, LCD). T0 represented the baseline of the entire observed nutritional pathway, whereas T1 represented the relevant baseline for evaluating changes occurring during the second-line KETOCOMP sequence.

The primary aim was to evaluate the effectiveness of a food-based sequential ketogenic nutritional protocol in patients who had previously failed to achieve clinically meaningful weight loss following a supervised hypocaloric Mediterranean diet.

Secondary aims were to evaluate longitudinal changes in BW, BMI, FM, WC, BCM, and PhA throughout the different phases of the nutritional pathway, as well as to assess the stability of anthropometric and body composition outcomes during the carbohydrate reintroduction phase.

The interval between T0 and T1 ranged from 40 to 300 days, reflecting the heterogeneity of routine outpatient clinical pathways. Some participants had already been followed with a hypocaloric MD before the ketogenic protocol became available as a therapeutic option in clinical practice. Additional variability was related to follow-up scheduling and individualized clinical decision-making. No predefined minimum duration of the Mediterranean diet phase was applied. T1 corresponded to the clinical visit at which the treating team evaluated the response to the MD and decided to initiate the ketogenic phase. In contrast, the ketogenic and carbohydrate-reintroduction phases had a fixed duration of four weeks each. The study protocol (KETOCOMP, Study ID 27798) was reviewed and approved by the Territorial Ethics Committee of Lazio Area 3 (Comitato Etico Territoriale Lazio Area 3, Rome, Italy): protocol number 895/26. The study was conducted in accordance with the principles of the Declaration of Helsinki.

### 2.2. Inclusion Criteria

The study population was considered representative of individuals with complicated obesity requiring a second-line nutritional intervention.

Participants were included if they met all the following criteria:

• Age ≥ 18 years.
• Diagnosis of obesity (BMI ≥ 30 kg/m^2^) or overweight (BMI ≥ 25 kg/m^2^) with metabolic complications.
• Completion of at least T0 and T1 assessments following a hypocaloric MD.
• Clinical identification of an insufficient or suboptimal response to the hypocaloric MD, leading to the decision to initiate ketogenic therapy, as operationally defined in Section 2.4.1. Availability of complete anthropometric and Bioelectrical Impedance Analysis-derived body composition data.
• Completion of the ketogenic phase, with evaluation at T2.
• Eligibility for the ketogenic phase confirmed by clinical assessment

### 2.3. Exclusion Criteria

Participants were excluded if they presented with any of the following:

• Medical contraindications to ketogenic diets.
• Pregnancy or breastfeeding.
• Documented poor adherence to the preceding hypocaloric Mediterranean diet sufficient to explain the inadequate clinical response, as recorded during routine follow-up.
• Missing primary anthropometric or body composition data.
• Premature interruption of the clinical pathway.
• Acute or unstable chronic diseases interfering with nutritional management or outcome interpretation.

The final cohort comprised 25 metabolically characterized patients who completed the nutritional protocol through T2; follow-up data at T3 were available for 21 participants.

### 2.4. Nutritional Intervention and Timeline

**Figure 1:**
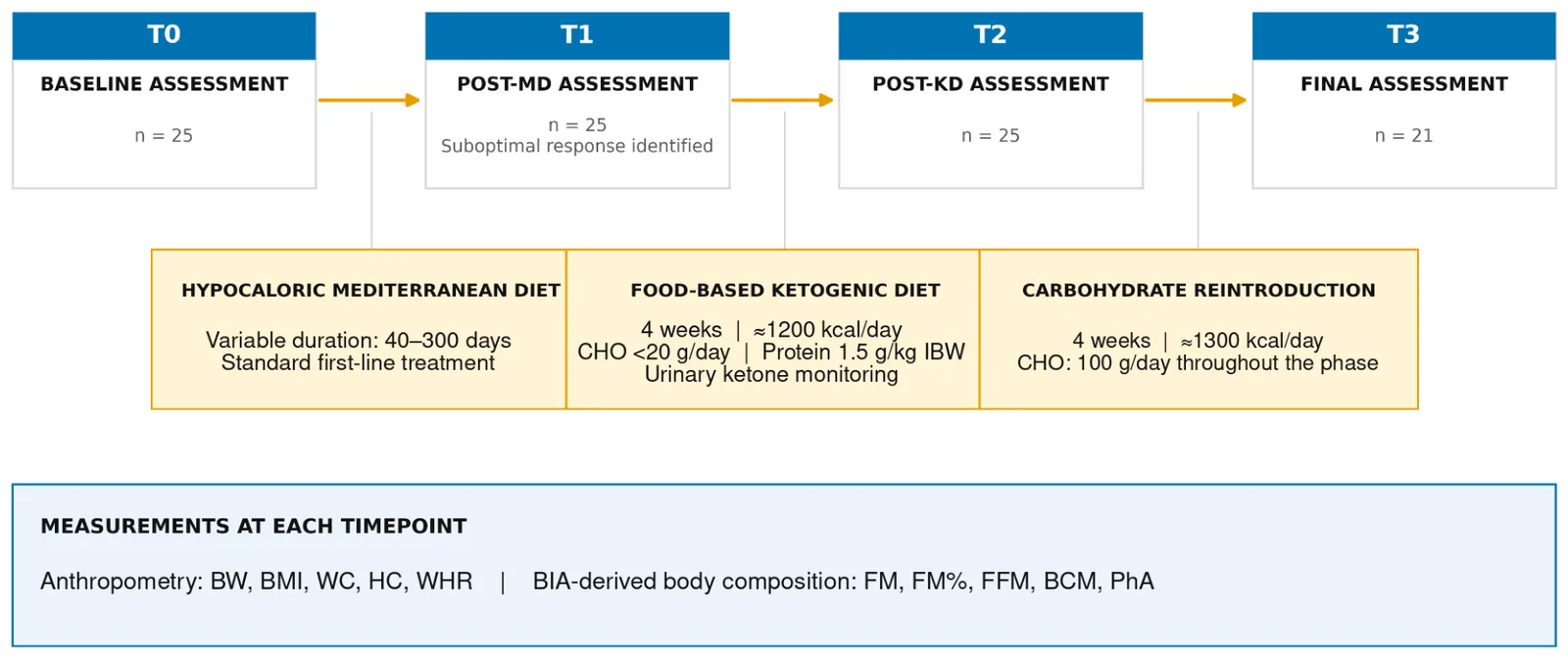
Study design and timeline of the KETOCOMP nutritional protocol. Participants underwent a hypocaloric Mediterranean diet of variable duration between T0 and T1, followed by a 4-week food-based ketogenic diet between T1 and T2 and a 4-week controlled carbohydrate reintroduction phase between T2 and T3. Anthropometric and BIA-derived body composition parameters were assessed at each timepoint. Nutritional ketosis was monitored during the ketogenic phase using urinary ketone testing.

The KETOCOMP protocol consisted of three sequential phases designed to reflect real-world outpatient practice. Table 1 summarizes the duration, intervention, and objective of each phase, while Figure 1 provides an integrated schematic overview of the study timeline, dietary treatments, measurement schedule, and nutritional ketosis monitoring.

#### 2.4.1. Mediterranean Diet Phase (T0–T1)

Participants followed a guideline-based hypocaloric MD as the standard initial intervention. The Mediterranean dietary plan was individually formulated on the basis of each patient’s medical and dietary history, sex, anthropometric and body composition characteristics, estimated basal metabolic rate and total energy requirements, physical activity level, metabolic comorbidities, and clinical nutritional needs. At T1, response to the hypocaloric MD was evaluated using a ≥5% reduction in baseline body weight as an objective benchmark for clinically meaningful weight loss. However, the decision to initiate the ketogenic phase was not based exclusively on this threshold and also considered the development of a plateau in body weight and/or BIA-derived fat mass, together with the treating team’s overall clinical assessment. Thus, patients could be considered to have an insufficient or suboptimal response despite having achieved the 5% weight-loss threshold if subsequent stabilization of body weight or fat mass, or their overall clinical condition, supported treatment intensification. This represented a pragmatic clinical assessment conducted during routine practice rather than a prospectively standardized study endpoint.

#### 2.4.2. Ketogenic Diet Phase (T1–T2)

A medically and dietitian-supervised, food-based KD was implemented for approximately 4 weeks. The dietary intervention provided a standardized energy intake of approximately 1200 kcal/day for all participants. This energy threshold was selected with reference to the clinical literature and guideline-based applications of ketogenic dietary interventions in obesity [65,66,67,68], as a less restrictive, entirely food-based alternative to conventional VLCKD protocols providing <800 kcal/day. The aim was to generate a clinically relevant energy deficit while allowing the provision of whole foods and adequate protein intake.

Basal metabolic rate and total energy requirements were estimated for each participant during the initial nutritional assessment and were used, together with the other individual clinical and anamnestic characteristics, to formulate the preceding hypocaloric Mediterranean dietary plan. During the subsequent ketogenic phase, these estimates continued to inform clinical assessment and monitoring but did not determine the caloric target, which remained standardized at approximately 1200 kcal/day. Individualization of the ketogenic diet primarily concerned protein intake, which was prescribed at approximately 1.5 g/kg of ideal body weight through one of four protein-adjusted dietary variants (P90–P120). Nutritional ketosis was promoted primarily through carbohydrate restriction to <20 g/day and was verified by urinary ketone monitoring.

Key features included:

• Protein intake: 1.5 g/kg ideal body weight, provided through natural foods and structured protein supplementation to support satiety and help limit excessive fat-free mass loss. Carbohydrates: Restricted to 20 g/day.
• Remaining energy: Supplied by predominantly mono- and polyunsaturated fats.

The nutrient composition of the standardized food-based dietary plans was calculated using the Italian Food Composition Database of the Council for Agricultural Research and Economics (CREA) [69].

Electrolyte and micronutrient supplementation was individualized according to each patient’s clinical and nutritional requirements, while soluble fiber supplementation was prescribed when indicated. Therefore, the nutrient composition reported for the dietary plans refers exclusively to food-based intake and does not include individualized supplementation.

Table 2 summarizes the macronutrient composition of the four protein-adjusted ketogenic variants, while their calculated fiber and selected mineral and vitamin contents are reported in Supplementary Table S1.

#### 2.4.3. Low-Carbohydrate Reintroduction Phase (T2–T3)

Following the ketogenic phase, participants transitioned directly to a 1300 kcal/day low-carbohydrate diet providing approximately 100 g of carbohydrates per day, which was maintained throughout the 4-week reintroduction phase. Unlike multi-phase reintroduction models described in VLCKD guidelines, this simplified single-step reintroduction (including whole grains, legumes, and fruits) was adopted for outpatient feasibility and adherence. Table 3 summarizes the macronutrient composition of the four protein-adjusted carbohydrate reintroduction variants, while their calculated fiber and selected mineral and vitamin contents are reported in Supplementary Table S2.

### 2.5. Monitoring and Measurements

All anthropometric and bioimpedance measurements were performed at T0, T1, T2, and T3 under standardized conditions to ensure inter-timepoint comparability.

#### 2.5.1. Anthropometric Measurements

Anthropometric assessments included: BW, BMI, WC, HC, and Waist-to-Hip Ratio (WHR). Measurements were taken with participants barefoot and in light clothing. WC and HC were measured using a non-elastic tape at standard anatomical landmarks.

#### 2.5.2. Body Composition Assessment

Body composition was assessed via Bioelectrical Impedance Vector Analysis (BIVA) using a BIA 101 BIVA PRO device. Measurements were performed in a supine position after 5–10 min rest, adhering to methodological recommendations for vector analysis. BIVA provides a direct, non-derivative assessment of hydration status and body compartment distribution. Primary BIVA-derived parameters included:

• BCM: A proxy for metabolically active tissue.
• PhA: A marker of cellular integrity and functional status.

The combined evaluation of BCM and PhA aimed to complement the assessment of quantitative weight loss by describing changes in bioelectrical indicators related to the metabolically active cellular compartment and cellular status.

#### 2.5.3. Assessment of Nutritional Ketosis

Nutritional ketosis was qualitatively monitored using urinary ketone detection (Ketostix™ colorimetric strips). This approach provided outpatient confirmation of ketosis onset and maintenance, without requiring quantitative ketone measurement for clinical decision-making.

#### 2.5.4. Clinical Tolerability and Adverse-Event Assessment

Clinical tolerability was evaluated as part of routine nutritional follow-up at the end of the 4-week ketogenic phase (T2) and again at the end of the carbohydrate-reintroduction phase (T3). Clinical interviews included the assessment of patient-reported symptoms potentially associated with the intervention, particularly headache, nausea or dizziness, fatigue or weakness, irritability, gastrointestinal symptoms, reduced exercise tolerance, and halitosis. When required, symptoms were managed through dietary counselling, adequate hydration, individualized electrolyte supplementation, and adjustment of supportive nutritional measures. Serious adverse events and premature discontinuations attributed to the dietary intervention were also assessed. Because this retrospective study was based on routine clinical practice, adverse events were not collected using a standardized questionnaire or prospective grading system, and the frequencies of individual symptoms could not be reliably quantified. Similarly, biochemical safety assessments were not performed according to a predefined schedule at each study timepoint. Laboratory tests were requested in selected patients according to individual clinical indications; therefore, these data were not sufficiently standardized or complete to be included as formal safety outcomes.

Moreover, because completion of the ketogenic phase through T2 was required for inclusion in the analytical cohort, the present study could not estimate the overall discontinuation rate among all patients who began the ketogenic phase.

### 2.6. Measurement Schedule

All anthropometric and body composition measurements were recorded at T0, T1, T2, and T3, with conditions standardized to ensure inter-timepoint comparability.

### 2.7. Statistical Analysis

Statistical analysis was conducted within an intra-subject framework, utilizing the R statistical environment to compare the evolution of measured parameters across time points (T0, T1, T2, and T3) for each individual. This approach aimed to minimize inter-individual variability and enhance the sensitivity in detecting changes induced by the nutritional intervention.

Because of the retrospective observational design, all consecutive eligible patients were included in the analysis. To assess whether the available sample size was adequate for the primary endpoint, a sample size evaluation was performed based on the within-subject change in body weight between T1 and T2. Assuming a clinically meaningful weight reduction of 3.0 kg, an estimated standard deviation of the paired differences of 3.1 kg, a two-sided α level of 0.05, and a statistical power of 80%, the minimum required sample size was calculated to be 9 participants. Since the final study population included 25 patients, the available sample size was considered adequate to detect the predefined primary outcome.

Prior to analysis, the normality of differences between time points for each variable was assessed using the Shapiro–Wilk test. Accordingly, paired *t*-tests were applied for variables exhibiting normal distributions, while Wilcoxon signed-rank tests were employed for those with non-normal distributions. Statistical significance for all analyses was set at *p* < 0.05. No adjustment for multiple comparisons was applied. Therefore, analyses of secondary outcomes should be considered exploratory and interpreted cautiously.

The variables subjected to analysis included both anthropometric and body composition parameters. Anthropometric variables comprised BW, BMI, WC, HC, and WHR. Body composition variables included absolute and relative FM, FFM, BCM and PhA. Among these, BW, FM, and WC were designated as primary endpoints due to their direct association with cardiometabolic risk reduction. BCM and PhA were classified as secondary functional endpoints, providing insight into the metabolic quality of weight loss rather than solely its quantitative extent.

Four distinct temporal windows were evaluated to assess changes throughout the protocol: T0 → T1, T1 → T2, T2 → T3, and T1 → T3. It is important to note that total body water compartments were monitored descriptively for hydro-electrolytic balance but were deliberately excluded from the inferential analysis, as they were not considered clinical outcomes of interest for statistical hypothesis testing.

## 3. Results

### 3.1. Baseline Characteristics

The cohort investigated in this study comprised 25 adult subjects, consisting of 16 women and 9 men, with a mean age of 48.3 ± 11.6 years. All participants presented with a diagnosis of obesity or overweight, further complicated by the presence of metabolic comorbidities. At the baseline assessment (T0), the participants exhibited a mean BMI of 34.4 ± 5.2 kg/m^2^. Additionally, elevated WHR values were observed within this group.

Analysis of body composition, which was performed using BIVA, indicated a mean FM of 40.2 ± 12.0 kg. Concurrently, values for BCM and PhA were recorded as lower than those reported in reference populations of metabolically healthy individuals.

The comprehensive baseline characteristics of the enrolled subjects are provided in Table 4.

### 3.2. Descriptive Trends in Anthropometric and Body Composition Parameters

**Figure 2:**
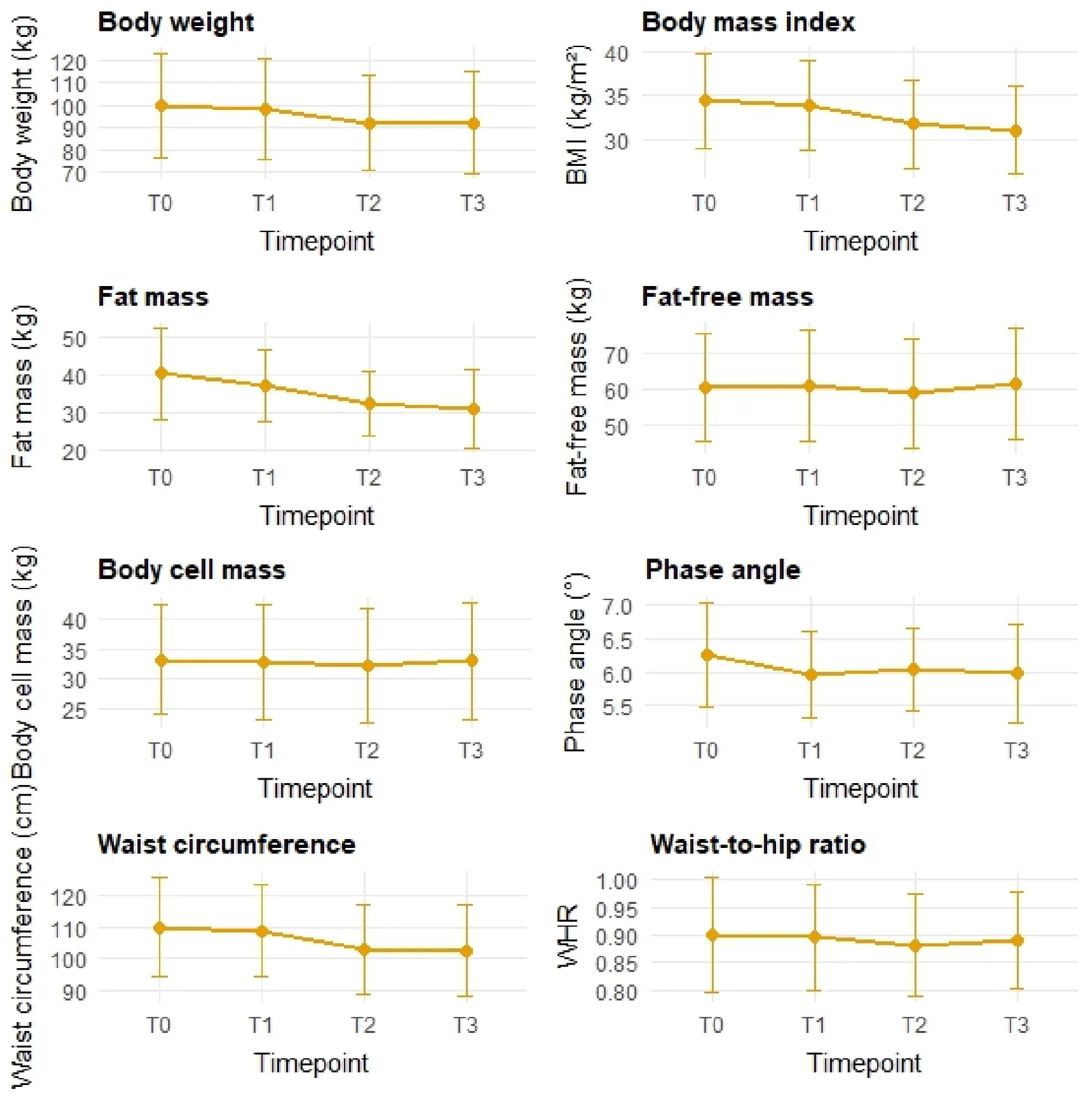
Temporal changes in anthropometric and body composition parameters across the intervention phases. BW, BMI, FM, FFM, BCM, PhA, WC and WHR are shown at baseline (T0), after hypocaloric MD (T1), after low-calorie KD (T2) and after LCD maintenance (T3). Data are expressed as mean ± standard deviation. Sample size was n = 25 at T0–T2 and n = 21 at T3 due to follow-up availability.

A descriptive analysis was conducted to examine the trajectories of anthropometric and compositional parameters across the four distinct time points of the study (T0, T1, T2, T3). The observed changes are presented in Table 5, with their temporal trends further illustrated in Figure 2.

#### 3.2.1. Weight and BMI

Between T0 and T3, mean BW decreased from 99.9 ± 22.9 kg to 92.3 ± 22.6 kg, corresponding to an overall decrease of −7.7%. The most pronounced reduction in BW was observed during the T1→T2 interval, which coincided with the ketogenic phase of the nutritional protocol. A further modest but statistically significant reduction in BW was observed during the subsequent low-carbohydrate reintroduction phase. Similarly, BMI progressively declined from 34.4 ± 5.2 kg/m^2^ at T0 to 31.2 ± 4.9 kg/m^2^ at T3, representing a −9.45% reduction.

#### 3.2.2. Body Composition

Absolute FM exhibited a steady decline throughout the protocol, decreasing from 40.2 ± 12.0 kg at T0 to 30.9 ± 10.3 kg at T3, marking a −23.1% reduction. The relative FM also decreased, moving from 39.3 ± 6.5% to 33.2 ± 6.3%.

FFM values were recorded as 60.4 ± 15.2 kg at T0 and 61.4 ± 15.5 kg at T3, indicating a consistent range.

BCM values were 33.2 ± 9.1 kg at T0 and 33.0 ± 9.6 kg at T3.

PhA measurements showed an overall decrease from 6.25 ± 0.78° to 5.98 ± 0.73° across the study period.

#### 3.2.3. Anthropometric Circumferences

WC decreased from 110.0 ± 15.6 cm at T0 to 102.7 ± 14.5 cm at T3, which corresponds to a −6.65% reduction. Similarly, HC decreased from 122.4 ± 13.1 cm to 115.3 ± 12.5 cm, representing a −5.80% reduction. WHR values remained stable throughout the observation period.

### 3.3. Inferential Analysis Across Nutritional Phases

To evaluate the statistical significance of the observed changes, inferential comparisons were conducted between consecutive phases (T0→T1, T1→T2, T2→T3) and for the entire ketogenic plus maintenance cycle (T1→T3). It should be noted that the initial phases (T0→T1, T1→T2) included 25 subjects. However, due to subject dropout between T2 and T3, analyses involving the T2→T3 and T1→T3 intervals were conducted on 21 subjects.

**Figure 3:**
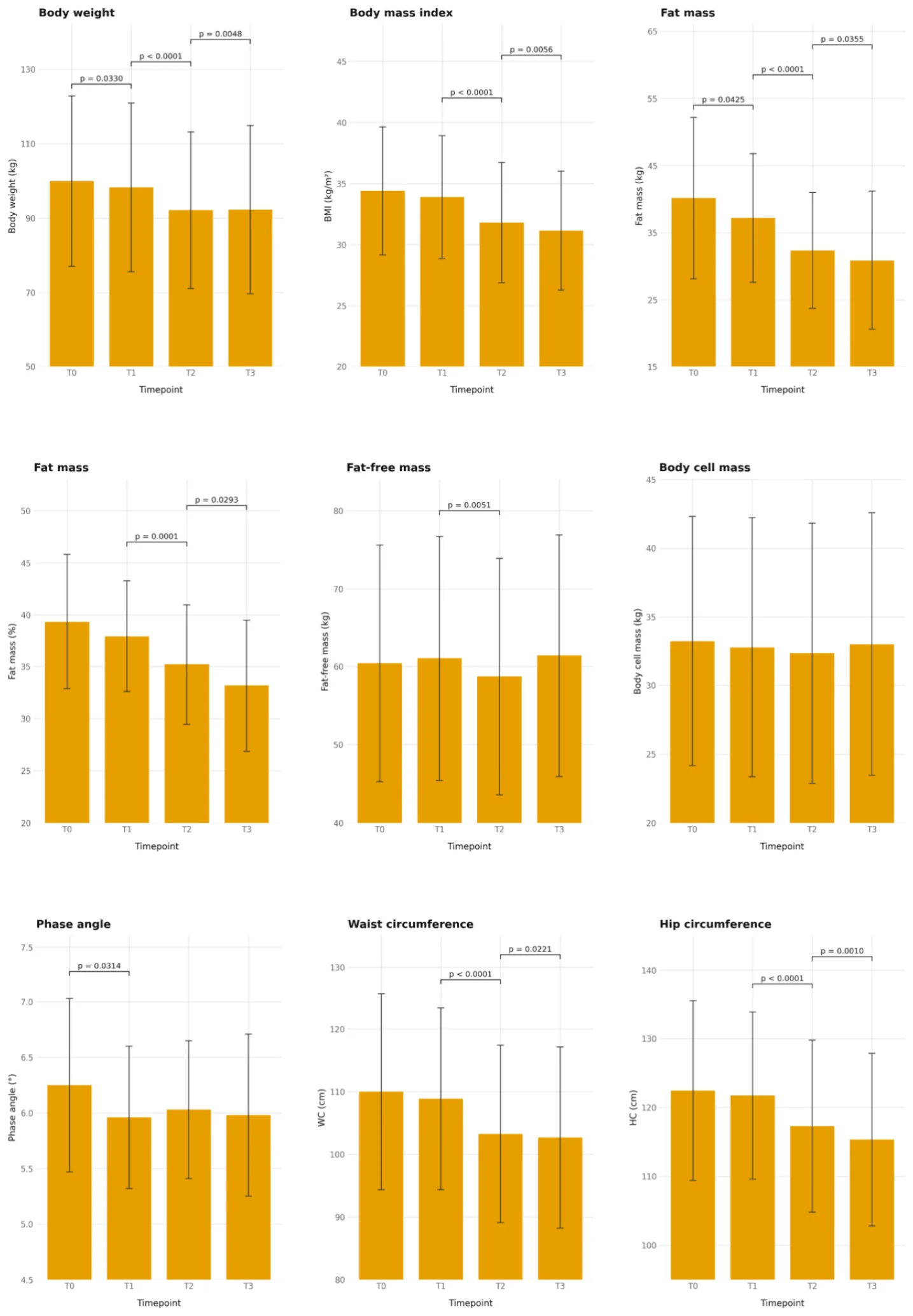
Anthropometric and body composition parameters across the intervention phases. Bars represent mean values and error bars indicate standard deviations at baseline (T0), after the hypocaloric Mediterranean diet (T1), after the low-calorie ketogenic diet (T2), and after the low-carbohydrate reintroduction phase (T3). Statistically significant pairwise comparisons between consecutive phases are indicated by brackets and corresponding *p*-values (*p* < 0.05); non-significant comparisons are not displayed. Sample size was n = 25 at T0–T2 and n = 21 at T3. Paired analyses involving T3 were conducted in the 21 participants with available follow-up data. For graphical clarity, y-axis ranges were adjusted separately for each variable and do not necessarily start at zero.

The results of these analyses are detailed in Table 6 and visually represented in Figure 3. Bar plots show mean values with standard deviation error bars, while statistically significant pairwise comparisons between consecutive phases are indicated by brackets and corresponding *p*-values.

#### 3.3.1. Mediterranean Diet Phase (T0→T1)

During the T0→T1 interval, BW (Δ = −1.68 kg, *p* = 0.0330), FM (Δ = −2.70 kg, *p* = 0.0425), and PhA (Δ = −0.29°, *p* = 0.0314) exhibited statistically significant reductions. No statistically significant changes were observed in BMI (Δ = −0.50 kg/m^2^, *p* = 0.1063), FFM (Δ = 0.63 kg, *p* = 0.3988), BCM (Δ = −0.45 kg, *p* = 0.4917), WC (Δ = −1.12 cm, *p* = 0.2748), HC (Δ = −0.72 cm, *p* = 0.3377), or WHR (Δ = 0.00, *p* = 0.5696).

#### 3.3.2. Ketogenic Diet Phase (T1→T2)

The T1→T2 interval demonstrated significant alterations across multiple parameters. BW (Δ = −6.1, *p* < 0.0001), BMI (Δ = −2.08, *p* < 0.0001), absolute FM (Δ = −4.8, *p* < 0.0001), relative FM (FM%, Δ = −1.9, *p* = 0.0001), WC (Δ = −5.62, *p* < 0.0001), and HC (Δ = −4.44, *p* < 0.0001) all decreased with high statistical significance. Additionally, FFM showed a statistically significant reduction (Δ = −1.4, *p* = 0.0051). BCM (Δ = −0.44, *p* = 0.3284) and PhA (Δ = 0.07, *p* = 0.4140) did not show statistically significant changes. The WHR also showed a statistically significant change (Δ = −0.01, *p* = 0.0401).

#### 3.3.3. Low-Carbohydrate Reintroduction Phase (T2→T3)

In the subsequent reintroduction phase (T2→T3), BW (Δ = −1.2, *p* = 0.0048) and BMI (Δ = −0.43, *p* = 0.0056) continued to decrease significantly. FFM (Δ = 0.2, *p* = 0.3602) and BCM (Δ = −0.16, *p* = 0.7924) showed no statistically significant changes. Furthermore, WC (Δ = −1.14, *p* = 0.0221) and HC (Δ = −2.29, *p* = 0.0010) demonstrated further statistically significant reductions, while PhA remained unchanged (Δ = −0.04, *p* = 0.6967). The WHR also showed no statistically significant change (Δ = 0.01, *p* = 0.0540).

#### 3.3.4. Ketogenic + Low-Carb Diet Phase (T1→T3)

The combined interval encompassing the ketogenic and reintroduction phases (T1→T3) showed significant reductions in BW (Δ = −7.79, *p* < 0.0001), BMI (Δ = −2.65, *p* < 0.0001), FM (Δ = −6.29, *p* < 0.0001), WC (Δ = −6.4, *p* < 0.0001), and HC (Δ = −6.57, *p* < 0.0001). A statistically significant decrease was also observed in FFM (Δ = −1.5, *p* = 0.0092). BCM (Δ = −0.75, *p* = 0.1922) and PhA (Δ = 0.02, *p* = 0.8395) exhibited no statistically significant changes. The WHR also showed no statistically significant change (Δ = 0, *p* = 0.5430).

### 3.4. Intra-Individual Response Patterns

**Figure 4:**
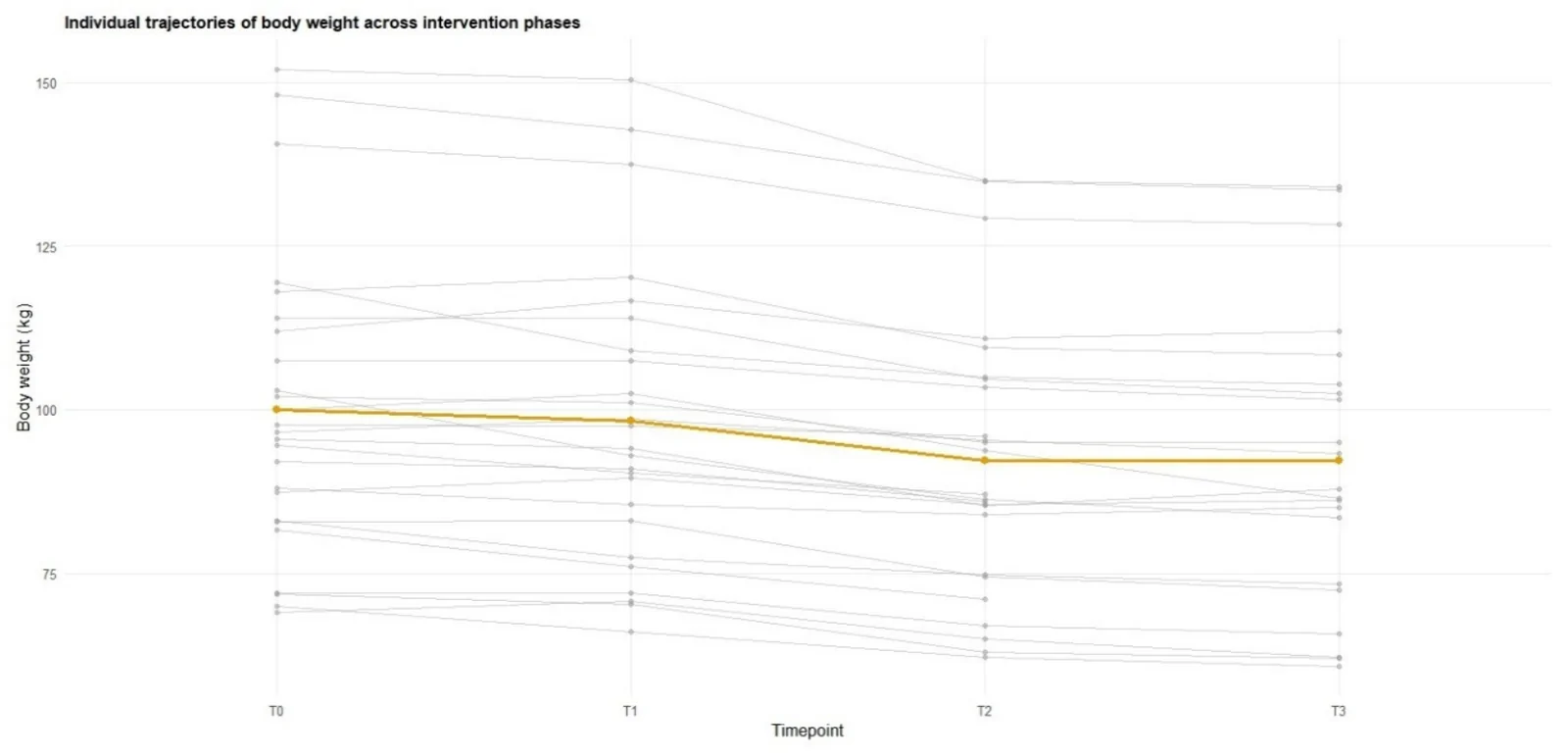
Individual trajectories of BW across the intervention phases (T0–T3). Grey lines represent individual participants, while the orange line and points indicate mean values at each timepoint. A consistent reduction in BW was observed in nearly all subjects during the ketogenic phase (T1→T2), followed by a smaller additional reduction during the carbohydrate-reintroduction phase (T2→T3). Sample size was 25 subjects up to T2 and 21 subjects at T3.

**Figure 5:**
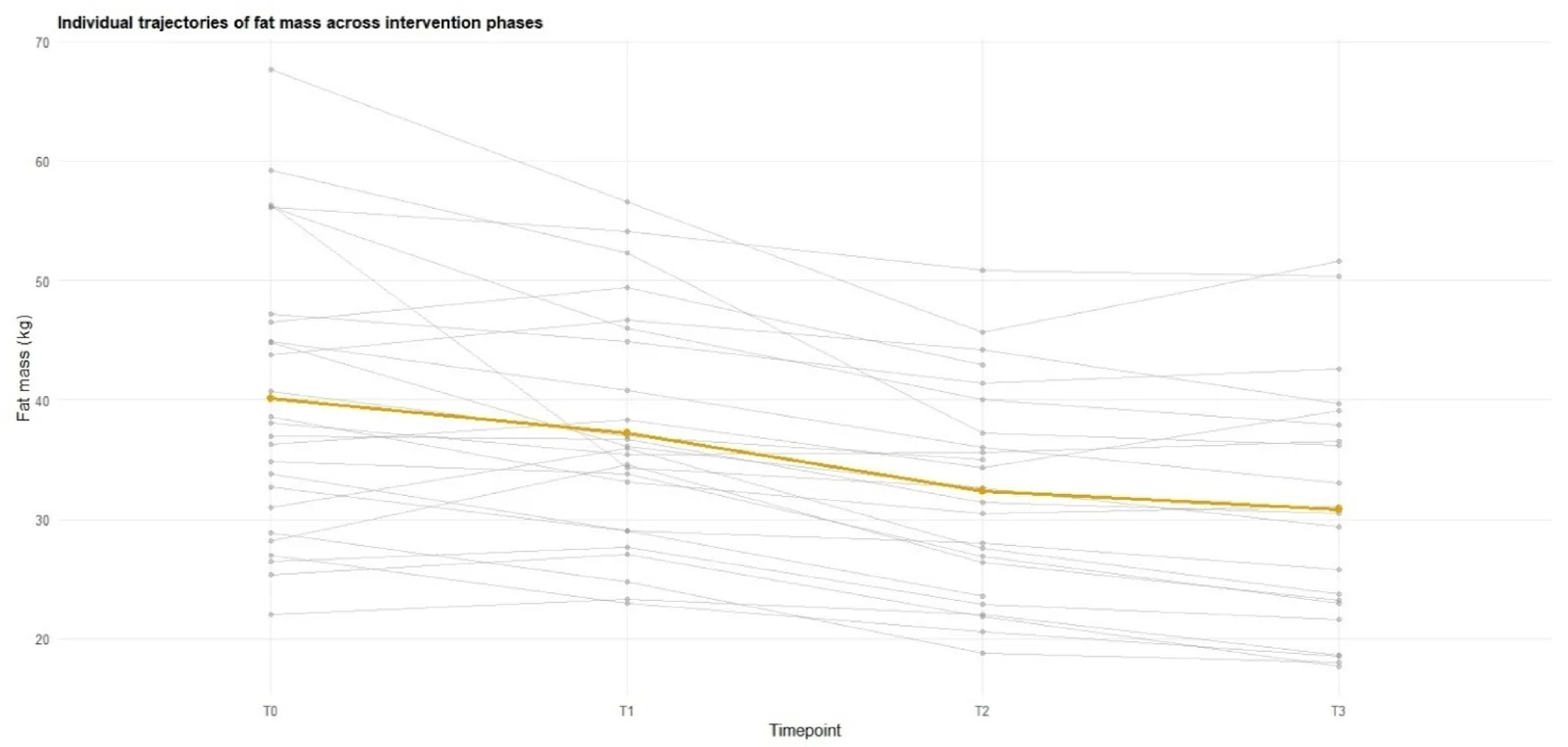
Individual trajectories of FM (kg) across the intervention phases (T0–T3). Grey lines represent individual participants, while the orange line and points indicate mean values at each timepoint. FM decreased consistently across subjects during the ketogenic phase (T1→T2), with reduced inter-individual variability, followed by a smaller but statistically significant additional reduction during the subsequent carbohydrate-reintroduction phase (T2→T3). Sample size was 25 subjects up to T2 and 21 subjects at T3.

Analysis of individual trajectories for BW and FM, depicted in Figure 4 and Figure 5, revealed consistent patterns across participants. A reduction in both BW and FM was observed for nearly all subjects between T1 and T2. During the ketogenic phase, the variability in individual trajectories decreased. During the low-carbohydrate reintroduction phase, BW and FM showed smaller additional reductions than during the ketogenic phase, although individual responses remained heterogeneous.

### 3.5. Composite Metabolic–Compositional Summary

**Figure 6:**
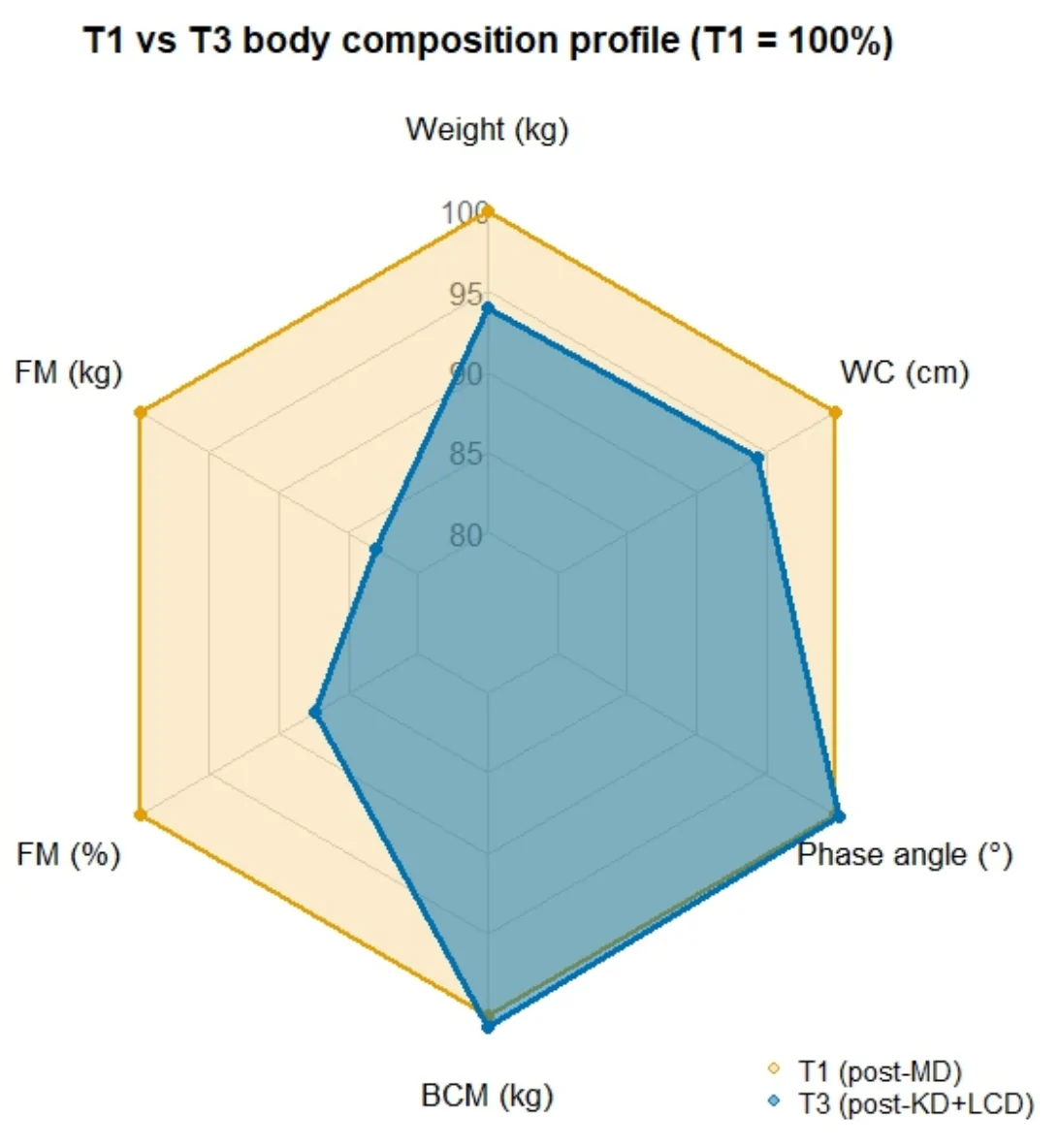
Normalized radar chart comparing body composition parameters at T1 and T3. Values at T1 were set to 100%, and values at T3 are expressed as a percentage of T1. Each axis represents a body composition variable (BW, FM [kg and %], BCM, PhA, and WC). Values below 100% indicate a reduction at T3 relative to T1.

To provide an integrated and synthetic representation of the effects of the nutritional pathway, a normalized radar chart (spider plot) was constructed (Figure 6). This graph visually compares the body composition profile of participants at two key time points: T1 (post–MD) and T3 (post–KD + LCD). In this plot, T1 values are normalized to 100%, and T3 values are expressed as a percentage of T1. Each axis of the radar represents one specific body composition variable: BW (kg), FM (kg), FM percentage—FM (%), BCM (kg), PhA (°), and WC (cm). The outer ring corresponds to 100%, while inner rings represent progressively lower percentages (e.g., 95%, 90%, 85%, 80%). Variables where the T3 polygon falls inside the T1 polygon indicate a reduction (such as BW, FM, and WC), while variables for which T3 remains close to the T1 reference line show comparatively small descriptive changes (e.g., BCM and PhA). The overall shape of the T3 polygon therefore reveals, at a glance, a global shift toward a healthier body composition after the ketogenic and low-carb reintroduction phases, illustrating reductions in BW, FM, and WC, together with comparatively small changes in BCM and PhA values.

### 3.6. Clinical Tolerability

No serious adverse events were documented among the 25 participants included in the analytical cohort. However, completion of the ketogenic phase through T2 formed part of cohort selection, while premature interruption of the clinical pathway was an exclusion criterion. Therefore, the present dataset does not provide the denominator or reasons for discontinuation among all patients who may have begun the ketogenic phase, and no overall diet-related discontinuation rate can be estimated. Four of the 25 included participants were unavailable for the final T3 assessment because they were lost to follow-up. Although potentially diet-related symptoms were assessed during routine clinical follow-up at T2 and T3, their individual frequencies and severity could not be reliably quantified from the retrospective records. Moreover, because biochemical monitoring was not performed according to a standardized prospective schedule, no comprehensive conclusions regarding biochemical safety can be drawn from the present dataset.

## 4. Discussion

Despite the widespread use of the MD as first-line therapy, robust evidence on effective and clinically applicable nutritional alternatives for patients classified as non-responders remains limited. The present study provides the first clinical evaluation of the KETOCOMP protocol, a food-based sequential ketogenic nutritional strategy specifically developed for Mediterranean diet non-responders with complicated obesity.

This study describes short-term anthropometric and BIA-derived body composition changes observed during a hypocaloric sequential ketogenic nutritional approach as a second-line strategy for individuals with obesity or MetS who demonstrated suboptimal responses to a hypocaloric MD, aligning with literature on subjects with metabolic adaptation or insulin resistance [70]. Rather than comparing ketogenic and Mediterranean dietary patterns, KETOCOMP was conceived as a structured second-line nutritional intervention for patients who fail to achieve clinically meaningful weight loss despite an adequately supervised hypocaloric Mediterranean diet.

Descriptive and inferential analyses revealed that modest and incomplete weight reductions during the initial MD phase were significantly improved by transitioning to the ketogenic model (T1→T2), which led to marked improvements in BW, anthropometric measures, and body composition. Further reductions were observed during the LCD phase (T2→T3). The changes observed during the ketogenic phase are compatible with the expected metabolic effects of severe carbohydrate restriction and nutritional ketosis. However, substrate oxidation and metabolic flexibility were not directly measured; therefore, no ketosis-specific mechanism can be established from the present findings.

A distinctive feature of the KETOCOMP protocol was the predominance of fat mass reduction within the observed weight loss. During the ketogenic phase (T1→T2), body weight decreased by 6.10 kg and absolute fat mass by 4.80 kg, while fat mass percentage also decreased significantly. These changes were substantially greater than those observed during the preceding hypocaloric Mediterranean diet phase, in which body weight and fat mass decreased by 1.68 and 2.70 kg, respectively. The additional reductions observed during carbohydrate reintroduction resulted in an overall T1→T3 decrease of 7.79 kg in body weight and 6.29 kg in fat mass. Taken together, these findings indicate that the response to KETOCOMP was not limited to a reduction in total body weight but involved a clinically relevant improvement in adiposity.

The effectiveness of the ketogenic phase may be explained by the combined effects of carbohydrate restriction, negative energy balance, and an adequate, individually prescribed protein intake. Severe carbohydrate restriction reduces circulating insulin levels and promotes the mobilization and oxidation of fatty acids, thereby facilitating the use of stored fat as an energy substrate [61,71,72,73]. Ketone bodies may also contribute to appetite control and improved dietary adherence, although these mechanisms were not directly assessed in the present study. Furthermore, the food-based design and close medical and dietitian supervision may have supported compliance and allowed protein intake, food selection, and clinical monitoring to be adapted to individual requirements. Therefore, the observed response probably reflects the combined action of nutritional ketosis, caloric restriction, protein adequacy, and structured clinical monitoring rather than ketosis alone.

Fat-free mass decreased by 1.40 kg during the ketogenic phase and by 1.50 kg across the complete T1→T3 interval. This finding should be interpreted cautiously because bioimpedance-derived fat-free mass includes body water and is influenced by changes in glycogen-associated water and fluid distribution that commonly occur during carbohydrate restriction [74]. Consequently, the observed reduction in fat-free mass cannot be interpreted exclusively as skeletal muscle loss. Importantly, body cell mass, which more closely reflects the metabolically active cellular compartment, did not change significantly during either the ketogenic phase or the complete sequential protocol. This divergence indicates that the significant reduction in bioimpedance-derived fat-free mass was not accompanied by a statistically significant change in body cell mass. However, the absence of direct measurements of skeletal muscle mass or muscle function precludes conclusions regarding skeletal muscle preservation.

Phase angle provides complementary information on cellular integrity, body cell mass, and hydration-related characteristics [75]. A small but statistically significant reduction was observed during the preceding Mediterranean diet phase (T0→T1), whereas phase angle remained stable during both the ketogenic phase and carbohydrate reintroduction. Its stability from T1 to T3, together with the absence of a significant reduction in body cell mass, suggests that the marked loss of body weight and fat mass was not accompanied by a detectable deterioration in the bioelectrical indicators of cellular status. Nevertheless, phase angle is an indirect marker influenced by hydration, and its nonsignificant change should be interpreted descriptively rather than as evidence of metabolically active tissue preservation.

Waist and hip circumferences decreased significantly during the ketogenic phase and continued to decline during carbohydrate reintroduction. Across T1→T3, waist circumference decreased by 6.40 cm and hip circumference by 6.57 cm. The reduction in waist circumference is clinically relevant because it represents a practical surrogate marker of central adiposity and cardiometabolic risk [3,73]. However, since visceral adipose tissue was not directly quantified by imaging, these findings should not be interpreted as direct evidence of selective visceral fat loss. The simultaneous reductions in waist circumference, hip circumference, absolute fat mass, and fat mass percentage nevertheless support a generalized improvement in adiposity and body fat distribution.

The controlled carbohydrate-reintroduction phase contributed to the consolidation of the response achieved during ketosis. Body weight, fat mass, waist circumference, and hip circumference continued to decrease between T2 and T3, while body cell mass and phase angle remained unchanged. This finding suggests that transitioning to a moderately carbohydrate-restricted diet did not produce an immediate reversal of the benefits obtained during the ketogenic phase. The sequential structure of KETOCOMP may therefore be clinically relevant: the ketogenic phase provides an intensive initial stimulus, whereas controlled carbohydrate reintroduction offers a more sustainable dietary pattern intended to support maintenance and outpatient adherence [65,66,76,77,78].

The normalized radar plot provides an integrated visual summary of these body composition changes across the complete KETOCOMP sequence (Figure 6). Compared with T1, the T3 polygon lies within the reference profile for adiposity-related variables, including body weight, absolute and relative fat mass, and waist circumference, visually reflecting their overall reduction. Conversely, body cell mass and phase angle remain close to the T1 reference values, consistently with their non-significant changes in the paired analyses. The radar plot should therefore be interpreted as a descriptive synthesis of the results rather than as an independent statistical analysis, complementing the detailed comparisons reported in Table 6 and Figure 3.

These findings align with extensive evidence on the efficacy of hypocaloric KD for complicated obesity, showing significant weight loss and improved glycemic and lipid profiles [61,65,70,79]. However, the current approach stands out for greater protein personalization and moderate caloric restriction. These features were intended to support nutritional adequacy and clinical tolerability during the intervention [63,80,81,82,83]. Although individualized protein intake may help limit excessive loss of lean tissue, the present findings cannot demonstrate prevention of muscle catabolism because skeletal muscle mass and muscle function were not directly assessed.

Changes in lean and metabolically active tissue are an important clinical consideration across different weight-loss interventions, including pharmacological treatments such as glucagon-like peptide 1 receptor agonists (GLP-RAs). Reductions in lean body mass have been reported during GLP-1-RA treatment, although their magnitude and clinical relevance vary according to the population, treatment, measurement method, and extent of overall weight loss (25–60% of total lost mass) [84,85,86,87,88,89,90]. In the present study, FFM decreased significantly during the complete KETOCOMP sequence, whereas BCM and PhA did not show statistically significant changes during the ketogenic and carbohydrate-reintroduction phases. However, these findings cannot demonstrate skeletal muscle preservation or establish a qualitative advantage over pharmacological treatments, because skeletal muscle mass and function were not directly assessed and no head-to-head comparison was performed. Rather than supporting comparative efficacy, these observations emphasize the need for future studies to evaluate the quality of weight loss using direct measures of muscle mass, strength, and physical performance across both nutritional and pharmacological obesity treatments.

In the ambulatory setting, these findings provide preliminary support for further investigation of supervised ketogenic nutritional interventions as second-line strategies for patients with an insufficient response to conventional hypocaloric regimens. However, their clinical applicability and generalizability require confirmation in larger prospective studies. The observed reactivation of weight loss and improvement in body composition, together with the absence of statistically significant changes in BIA-derived BCM, a proxy for metabolically active tissue, support further investigation of the protocol as a potential component of the integrated clinical management of dysmetabolic obesity, alongside educational programs and structured nutritional follow-up [84,89,90,91].

The present study should not be interpreted as a superiority trial comparing ketogenic diets with Mediterranean diets. Rather, it evaluates the effectiveness of a sequential ketogenic nutritional protocol in a clinically selected subgroup of patients considered to have an insufficient or suboptimal response to a supervised hypocaloric Mediterranean diet. Therefore, the observed benefits should be interpreted as the effect of a second-line nutritional strategy in metabolically or clinically non-responsive individuals, rather than evidence supporting the superiority of one dietary pattern over another.

No serious adverse events were documented among participants included in the analytical cohort. Nevertheless, because completion through T2 formed part of cohort selection, the study cannot estimate the discontinuation rate among all patients who began the ketogenic phase, nor can the absence of discontinuations within the selected cohort be interpreted as evidence of tolerability or as a comprehensive demonstration of safety. Although potentially diet-related symptoms were evaluated during routine follow-up at T2 and T3, their frequency and severity could not be reliably quantified because adverse events were not collected using a standardized prospective instrument. In addition, biochemical safety parameters were not available according to predefined timepoints for all participants. Therefore, the present findings provide information on short-term clinical tolerability but cannot establish the biochemical or longer-term safety of the KETOCOMP protocol.

The present findings should be interpreted in light of several limitations, including the retrospective single-arm design, small sample size, and relatively short follow-up. By design, the cohort excluded patients whose insufficient response to the preceding hypocaloric Mediterranean diet was considered attributable to documented poor adherence, as well as patients with missing primary outcome data or who interrupted the clinical pathway before completing the ketogenic phase. Although this selection was consistent with the aim of evaluating patients with an insufficient or suboptimal response despite adequate dietary adherence, it may have introduced selection bias and limited the generalizability of the observed outcomes and short-term clinical tolerability to broader clinical populations. In addition, four participants were lost to follow-up before T3, preventing assessment of outcomes after carbohydrate reintroduction in the complete cohort.

The absence of an isoenergetic non-ketogenic control group prevents separation of the effects of carbohydrate restriction and nutritional ketosis from those of the concurrent energy deficit. Moreover, the standardized 1200 kcal/day prescription resulted in energy deficits of different relative magnitude according to individual energy requirements. Skeletal muscle mass and muscle function were not directly assessed using imaging-based methods, muscle strength testing, or physical performance measures; therefore, the stability of BCM and PhA cannot be interpreted as definitive evidence of skeletal muscle preservation. Adverse-event assessment was based on routine clinical documentation rather than on a standardized prospective questionnaire or grading system, and the frequency and severity of individual symptoms could not be reliably quantified. Biochemical safety parameters were also not systematically available at predefined timepoints. Consequently, the study was not designed to provide a comprehensive assessment of biochemical or longer-term safety. In addition, no adjustment for multiplicity was applied despite the number of secondary comparisons performed, increasing the possibility of type I error; consequently, findings for secondary outcomes should be considered exploratory. Nevertheless, the within-subject design and detailed bioimpedance assessment provide preliminary real-world evidence supporting further investigation of the protocol in larger prospective controlled studies. Given the exploratory nature of this real-world observational study and the limited sample size, the statistical analysis was intentionally based on predefined within-subject pairwise comparisons rather than repeated-measures modeling. This approach was selected to evaluate clinically meaningful transitions between consecutive phases of the KETOCOMP protocol while minimizing inter-individual variability.

Future prospective randomized studies should evaluate whether the KETOCOMP protocol can improve long-term weight maintenance, body composition, and cardiometabolic outcomes compared with other evidence-based nutritional approaches.

Future directions also include direct comparisons with next-generation pharmacological strategies, focusing on the quality of weight loss and lean mass preservation, and investigating the KD’s impact on gut microbiota. KDs can alter microbiome composition, with some evidence suggesting decreased bacterial diversity and reduced short-chain fatty acids [92,93,94,95,96,97,98], though specific formulations may induce differentiated, potentially positive systemic outcomes [95,96,99,100,101,102]. Exploring how this protocol influences the gut microbiota and its contribution to observed metabolic effects remains a pertinent area for investigation [103,104].

## 5. Conclusions

This study addressed the clinical challenge of managing obesity and its metabolic complications, particularly focusing on patients who respond inadequately to conventional dietary approaches, such as the hypocaloric MD. We proposed and evaluated an innovative sequential nutritional protocol, consisting of a food-based ketogenic phase followed by controlled carbohydrate reintroduction.

Our findings suggest that this approach may produce clinically relevant short-term reductions in body weight, fat mass, and body circumferences. Although FFM decreased significantly, BCM and PhA did not show significant changes during the ketogenic and carbohydrate-reintroduction phases. These findings suggest that the observed weight loss was predominantly associated with a reduction in fat mass and was not accompanied by detectable deterioration in the measured bioelectrical indicators of cellular status. However, in the absence of direct measurements of skeletal muscle mass and function, no definitive conclusions regarding muscle preservation can be drawn. The additional reductions observed during carbohydrate reintroduction indicate that the improvements achieved during the ketogenic phase were not immediately reversed over the subsequent four weeks, but longer follow-up is required to assess their maintenance and sustainability.

In conclusion, a supervised, food-based sequential ketogenic protocol may represent a clinically applicable second-line nutritional strategy for patients with obesity and metabolic complications who respond suboptimally to conventional dietary treatment. KETOCOMP should be regarded as an integrated sequential intervention combining a ketogenic phase with controlled carbohydrate reintroduction rather than as a ketogenic diet alone. Its effectiveness, biochemical and longer-term safety, reproducibility, and durability should be evaluated in larger prospective controlled studies incorporating standardized adverse-event monitoring and direct assessment of skeletal muscle mass and function.

## Figures and Tables

**Table 1:** Nutritional pathway timeline.

Phase	Interval	Intervention	Objective
T0–T1	Variable (40–300 days)	Hypocaloric MD	Initial weight reduction
T1–T2	4 weeks	Food-based KD	Carbohydrate restriction and induction of nutritional ketosis
T2–T3	4 weeks	LCD with CHO reintroduction	Controlled carbohydrate reintroduction and short-term consolidation

Abbreviations: MD, Mediterranean diet; KD, ketogenic diet; LCD, low carbohydrate reintroduction diet; CHO, carbohydrates.

**Table 2:** Macronutrient composition of ketogenic diet variants (T1–T2).

Variant	kcal/Day	Protein (g)	Carbohydrates (g)	Lipids (g)
P90	1200	90	20	85.0
P100	1200	100	20	80.6
P110	1200	110	20	76.1
P120	1200	120	20	71.7

**Table 3:** Macronutrient composition during carbohydrate reintroduction (T2–T3).

Variant	kcal/Day	Protein (g)	Carbohydrates (g)	Lipids (g)
P90	1300	90	100	62.8
P100	1300	100	100	58.3
P110	1300	110	100	53.9
P120	1300	120	100	49.4

**Table 4:** Baseline characteristics of enrolled subjects, values expressed as mean ± standard deviation.

Variable	Unit of Measure	Mean ± SD/Distribution
Sex	*n*	M: 9 (36%); F: 16 (64%)
Age	years	48.3 ± 11.6
BW	kg	99.9 ± 22.9
BMI	kg/m^2^	34.4 ± 5.2
WC	cm	110.0 ± 15.6
HC	cm	122.4 ± 13.0
WHR	-	0.90 ± 0.10
FM	kg	40.2 ± 12.0
FM	%	39.3 ± 6.5
FFM	kg	60.4 ± 15.2
BCM	kg	33.2 ± 9.1
PhA	°	6.3 ± 0.8

Abbreviations: SD, standard deviation; M, Male; F, Female; BW, Body Weight; BMI, Body Mass Index; WC, Waist Circumference; HC, Hip Circumference, WHR, Waist-to-Hip Ratio; FM, Fat Mass; FFM, Fat-Free Mass; BCM, Body Cell Mass; PhA, Phase Angle.

**Table 5:** Descriptive trend of anthropometric and bioimpedance parameters; values expressed as mean ± standard deviation. Δ% indicates the descriptive percentage change between T3 and the T0 baseline of the entire observed nutritional pathway; T1 represents the baseline of the second-line KETOCOMP sequence.

Variable	Unit of Measure	T0 (*n* = 25)	T1 (*n* = 25)	T2 (*n* = 25)	T3 (*n* = 21)	Δ%
BW	kg	99.94 ± 22.88	98.26 ± 22.69	92.15 ± 21.03	92.28 ± 22.60	−7.66
BMI	kg/m^2^	34.40 ± 5.24	33.90 ± 5.02	31.81 ± 4.92	31.15 ± 4.86	−9.45
FM	kg	40.15 ± 12.01	37.20 ± 9.59	32.36 ± 8.63	30.86 ± 10.34	−23.14
FM %	%	39.34 ± 6.47	37.94 ± 5.33	35.21 ± 5.76	33.18 ± 6.31	−15.66
FFM	kg	60.43 ± 15.17	61.06 ± 15.65	58.73 ± 15.16	61.42 ± 15.48	1.63
BCM	kg	33.24 ± 9.07	32.79 ± 9.43	32.34 ± 9.47	33.02 ± 9.56	−0.65
PhA	°	6.25 ± 0.78	5.96 ± 0.64	6.03 ± 0.62	5.98 ± 0.73	−4.41
WC	cm	110.00 ± 15.63	108.88 ± 14.51	103.26 ± 14.17	102.69 ± 14.46	−6.65
HC	cm	122.44 ± 13.05	121.72 ± 12.13	117.28 ± 12.48	115.33 ± 12.52	−5.80
WHR	-	0.90 ± 0.10	0.90 ± 0.10	0.88 ± 0.09	0.89 ± 0.09	−1.05

Abbreviations: T0, Baseline; T1, Post-Mediterranean Diet Phase; T2, Post-Ketogenic Diet Phase; T3, Post-Low-Carbohydrate Reintroduction Phase; BW, Body Weight, BMI, Body Mass Index; FM, Fat Mass; FFM, Fat-Free Mass; BCM, Body Cell Mass; PhA, Phase Angle; WC, Waist Circumference; HC, Hip Circumference; WHR, Waist-to-Hip Ratio; Δ%, Percentage Change.

**Table 6:** Inferential analysis of within-subject changes across the different phases of the nutritional protocol (T0, T1, T2 and T3). Paired *t*-tests or Wilcoxon signed-rank tests were applied according to data distribution. Statistically significant results are highlighted in bold (*p* < 0.05).

Variable	Δ T0→T1	*p* T0→T1	Test T0→T1	n T0→T1	Δ T1→T2	*p* T1→T2	Test T1→T2	n T1→T2	Δ T2→T3	*p* T2→T3	Test T2→T3	n T2→T3	Δ T1→T3	*p* T1→T3	Test T1→T3	n T1→T3
BW (kg)	−1.68	**0.0330**	Paired *t*-test	25	−6.1	**<0.0001**	Paired *t*-test	25	−1.2	**0.0048**	Wilcoxon	21	−7.79	**<0.0001**	Paired *t*-test	21
BMI (kg/m^2^)	−0.5	0.1063	Paired *t*-test	25	−2.08	**<0.0001**	Paired *t*-test	25	−0.43	**0.0056**	Paired *t*-test	21	−2.65	**<0.0001**	Paired *t*-test	21
FM (kg)	−2.7	**0.0425**	Wilcoxon	25	−4.8	**<0.0001**	Wilcoxon	25	−2.1	**0.0355**	Wilcoxon	21	−6.29	**<0.0001**	Wilcoxon	21
FM (%)	−1.4	0.0525	Paired *t*-test	25	−1.9	**0.0001**	Wilcoxon	25	−1.31	**0.0293**	Paired *t*-test	21	−4.01	**<0.0001**	Paired *t*-test	21
FFM (kg)	0.63	0.3988	Paired *t*-test	25	−1.4	**0.0051**	Wilcoxon	25	0.2	0.3602	Wilcoxon	21	−1.5	**0.0092**	Paired *t*-test	21
BCM (kg)	−0.45	0.4917	Paired *t*-test	25	−0.44	0.3284	Paired *t*-test	25	−0.16	0.7924	Paired *t*-test	21	−0.75	0.1922	Paired *t*-test	21
PhA	−0.29	**0.0314**	Paired *t*-test	25	0.07	0.4140	Paired *t*-test	25	−0.04	0.6967	Paired *t*-test	21	0.02	0.8395	Paired *t*-test	21
WC (cm)	−1.12	0.2748	Paired *t*-test	25	−5.62	**<0.0001**	Paired *t*-test	25	−1.14	**0.0221**	Paired *t*-test	21	−6.4	**<0.0001**	Paired *t*-test	21
HC (cm)	−0.72	0.3377	Paired *t*-test	25	−4.44	**<0.0001**	Paired *t*-test	25	−2.29	**0.0010**	Paired *t*-test	21	−6.57	**<0.0001**	Paired *t*-test	21
WHR	0	0.5696	Paired *t*-test	25	−0.01	**0.0401**	Paired *t*-test	25	0.01	0.0540	Paired *t*-test	21	0	0.5430	Paired *t*-test	21

Abbreviations: BW, body weight; BMI, body mass index; BCM, body cell mass; FM, fat mass; FFM, fat-free mass; PhA, phase angle; WC, waist circumference; HC, hip circumference; WHR, waist-to-hip ratio; Δ, absolute change between timepoints; T0, baseline; T1, after hypocaloric Mediterranean diet; T2, after low-calorie ketogenic diet; T3, after low-carbohydrate maintenance.

## Data Availability

The data presented in this study are available from the corresponding author upon reasonable request. The data are not publicly available due to privacy and ethical restrictions related to participant confidentiality.

## Supplementary Materials

The following supporting information can be downloaded at: https://www.mdpi.com/article/10.3390/nu18182982/s1, Table S1: Calculated fiber and selected mineral and vitamin composition of the standardized food-based ketogenic dietary plans; Table S2: Calculated fiber and selected mineral and vitamin composition of the standardized food-based carbohydrate reintroduction dietary plans.

## Abbreviations

The following abbreviations are used in this manuscript:

BHB	β-Hydroxybutyrate
BIA	Bioelectrical Impedance Analysis
BIVA	Bioelectrical Impedance Vector Analysis
BCM	Body Cell Mass
BMI	Body Mass Index
BW	Body Weight
CHO	Carbohydrate
CVD	Cardiovascular Disease
FFM	Fat-Free Mass
FM	Fat Mass
GLP-1 RA	Glucagon-Like Peptide-1 Receptor Agonist
HC	Hip Circumference
IL-6	Interleukin-6
KD	Ketogenic Diet
LCD	Low-Carbohydrate reintroduction Diet
LGCI	Low-Grade Chronic Inflammation
MASLD	Metabolic Dysfunction-Associated Steatotic Liver Disease
MD	Mediterranean Diet
MetS	Metabolic Syndrome
MHO	Metabolically Healthy Obesity
MUO	Metabolically Unhealthy Obesity
NLRP3	NOD-like receptor protein 3
PhA	Phase Angle
SD	Standard Deviation
T2DM	Type 2 Diabetes Mellitus
TNF-α	Tumor Necrosis Factor Alpha
VLCKD	Very Low-Calorie Ketogenic Diet
WC	Waist Circumference
WHR	Waist-to-Hip Ratio
