# Supplementary material for: A Food-Based Sequential KETOgenic Nutritional Protocol for Mediterranean Diet Non-Responders with COMPlicated Obesity: A Real-World Observational Study (KETOCOMP)
Source: Nutrients. 2026 Sep 11;18(18):2982. doi: 10.3390/nu18182982 (PMC13610975; doi:10.3390/nu18182982)
Supplement: Supplementary file 1 [file nutrients-18-02982-s001.zip › nutrients-4515058-supplementary.pdf]

Supplementary Table S1. Calculated fiber and selected mineral and vitamin composition of the standardized food-based ketogenic dietary plans.

Variant	DF (g)	Na (mg)	K (mg)	Fe (mg)	Ca (mg)	P (mg)	Vit B1 (mg)	Vit B2 (mg)	Vit B3 (mg)	Vit A (μg RE)	Vit C (mg)	Vit E (mg)
P90	16.14	440.4	2665	5.13	278.7	911.6	0.707	1.384	17.38	55.7	80.5	17.52
P100	16.14	456.9	2850	5.32	280.7	1016.6	0.757	1.484	21.53	53.9	80.5	16.40
P110	15.79	456.6	2850	5.06	267.6	992.8	0.699	1.467	21.45	53.3	80.5	16.40
P120	15.79	456.6	2850	5.05	267.6	992.8	0.699	1.467	21.45	51.5	80.5	15.28

Abbreviations: DF, dietary fiber; Na, sodium; K, potassium; Fe, iron; Ca, calcium; P, phosphorus; Vit B1, thiamine; Vit B2, riboflavin; Vit B3, niacin; Vit A, vitamin A; RE, retinol equivalents; Vit C, vitamin C; Vit E, vitamin E; P90–P120, protein-adjusted ketogenic dietary variants.

Note: Nutrient values were calculated using the Italian Food Composition Database of the Council for Agricultural Research and Economics (CREA) and refer exclusively to the standardized dietary plans. The table reports the fiber and selected micronutrient data available from the dietary calculation worksheet and does not represent a complete micronutrient profile. Individually prescribed electrolyte, micronutrient, and soluble fiber supplements were not included.

Supplementary Table S2. Calculated fiber and selected mineral and vitamin composition of the standardized food-based carbohydrate reintroduction dietary plans.

Variant	DF (g)	Na (mg)	K (mg)	Fe (mg)	Ca (mg)	P (mg)	Vit B1 (mg)	Vit B2 (mg)	Vit B3 (mg)	Vit A (μg RE)	Vit C (mg)	Vit E (mg)
P90	18.89	904.1	2383	5.32	438.1	1031.8	0.781	1.667	16.685	247.1	132.5	9.516
P100	18.89	920.6	2568	5.51	440.1	1136.8	0.831	1.767	20.835	245.3	132.5	8.396
P110	18.54	920.3	2568	5.25	427.0	1113.0	0.773	1.750	20.755	244.7	132.5	8.396
P120	18.54	920.3	2568	5.24	427.0	1113.0	0.773	1.750	20.755	242.9	132.5	7.276

Abbreviations: DF, dietary fiber; Na, sodium; K, potassium; Fe, iron; Ca, calcium; P, phosphorus; Vit B1, thiamine; Vit B2, riboflavin; Vit B3, niacin; Vit A, vitamin A; RE, retinol equivalents; Vit C, vitamin C; Vit E, vitamin E; P90–P120, protein-adjusted carbohydrate reintroduction dietary variants.

Note: Nutrient values were calculated using the Italian Food Composition Database of the Council for Agricultural Research and Economics (CREA) and refer exclusively to the standardized dietary plans. The table reports the fiber and selected micronutrient data available from the dietary calculation worksheet and does not represent a complete micronutrient profile. Individually prescribed electrolyte, micronutrient, and soluble fiber supplements were not included.
